# A homozygous missense variant, p.Ser166Leu in the *PRPF40B* gene, in a 7.7 Mb region of homozygosity in a consanguineous Turkish family with essential tremor

**DOI:** 10.3389/fneur.2026.1796664

**Published:** 2026-05-26

**Authors:** Onur Emre Onat, Shilpa Sonti, Daphne Robakis, Ayse B. Tekinay, Cenk Akbostanci, F. Nazli Durmaz Celik, Tayfun Ozcelik, Lorraine N. Clark

**Affiliations:** 1 Department of Molecular Biology and Genetics, Bilkent University, Ankara, Türkiye; 2 Department of Pathology and Cell Biology, College of Physicians and Surgeons, Columbia University, New York, NY, United States; 3 Taub Institute for Research on Alzheimer’s Disease and the Aging Brain, College of Physicians and Surgeons, Columbia University, New York, NY, United States; 4 Department of Neurology, Yale School of Medicine, Yale University, New Haven, CT, United States; 5 Institute of Materials Science and Nanotechnology, Bilkent University, Ankara, Türkiye; 6 Department of Neurology, Faculty of Medicine, Ankara University, Ankara, Türkiye; 7 Department of Neurology, Faculty of Medicine, Osmangazi University, Eskisehir, Türkiye

**Keywords:** consanguinity, essential tremor, neurological disorder, Parkinson’s disease, tremor, Turkish, nuclear speckle protein, PRPF40B

## Abstract

**Introduction:**

We leveraged consanguinity and population endogamy in a large four generation Turkish family with ET. Examination of clinical features and genetic analysis identified a homozygous *PRPF40B* missense variant, in a large region of homozygosity, segregating with ET in the family.

**Methods:**

The ET family is of Turkish origin. The proband and relatives were evaluated at Ankara University Medical School and Bilkent University (Ankara, Turkey). A second evaluation of the clinical and videotape data was performed at Yale University. A total of 6 individuals from the family were clinically assessed. Whole genome sequencing and autozygosity mapping was performed in affected and unaffected family members. Transient transfection of HEK293T cells was performed to assess pathogenicity of the *PRPF40B* missense variant.

**Results:**

A total of 4 family members were diagnosed with ET; two individuals also had a diagnosis of Parkinson’s Disease (PD). We identified a missense variant in *PRPF40B*, p.Ser166Leu, located within a 7.7 Mb region of homozygosity (ROH) (Chr12: 46595701–54,330,441), which was observed in all analyzed affected family members. Unlike wildtype *PRPF40B* which localizes to nuclear speckles in the nuclear compartment, when mutant *PRPF40B* was expressed in HEK293T cells we observed enrichment and localization to the nuclear membrane.

**Discussion:**

The *PRPF40B* p.Ser166Leu variant is located within a conserved region of the WW protein domain. *In Silico* pathogenicity and functional studies predict that the missense variant alters the function of *PRPF40B*. *PRPF40B* has previously been implicated in the pathogenesis of neurological disorders, including Huntington’s disease and Rett syndrome.

## Introduction

Essential Tremor (ET) is one of the most common neurological disorders worldwide (1). The defining clinical feature of ET is a 4–12 Hz kinetic tremor of the hands during voluntary movement. Tremor may also occur in the head and voice, and 30–60% of ET patients develop a head tremor (2). Estimates of the global prevalence rates of ET, from 540,558 participants (pooled from 42 studies) suggest 1.33% (95% CI: 0.88–2.02%) of people are affected (3). Prevalence increases with age and for individuals aged 65 and older the total prevalence of ET is estimated to be 5.79% (95% CI:4.14–8.05%) (4).

In Turkey, door-to-door studies of prevalence estimates for ET range from 1.6% in Erzurum (5), 3.09% in Sile (6), and 4% in Mersin province (7) for people between the ages of 18–60, suggesting a slightly higher prevalence of ET in Turkey.

Consanguineous marriage is frequent in Turkey. Although there are regional differences, in Eastern Turkey the frequency of consanguineous marriage is as high as 34.4% (8). This high rate of consanguinity has facilitated disease gene discovery and led to the identification of many disease genes associated with neurological disorders in Turkey (9–11). In a large six-generation Turkish kindred with both ET and Parkinson’s Disease (PD), a missense variant (p.G399S) in the *HTRA2* gene was identified. ET was present in individuals heterozygous or homozygous for the *HTRA2* p.G399S allele and homozygosity was associated with an earlier age at onset (*p* < 0.0001) and disease severity (*p* < 0.0001) (11). *HTRA2* encodes a serine protease localized to mitochondria and is released into the cytosol following apoptotic stimuli (12). *HTRA2* proteolytic activity also triggers caspase-independent cell death (13).

In this study, we leveraged consanguinity and population endogamy in a cohort of Turkish families with ET. In one large four-generation family, with multiple affected family members segregating ET, we examined their clinical features and carried out whole-genome sequencing and autozygosity mapping to identify the genetic basis of ET in this family.

## Methods

### Ethics

Study subjects and relatives were enrolled in a Turkish family study of ET at Columbia University (NY, United States), Yale University (CT, United States), and Bilkent University (Ankara, Turkey). The Institutional Review Board approved the study at each institution, and a signed informed consent was obtained from all participants.

### Subjects

Family TR-ETM-54 is of Turkish origin. The proband and relatives were evaluated at Ankara University Medical School and Bilkent University (Ankara, Turkey). A movement disorder neurologist at Yale University (Dr. Daphne Robakis) performed a second evaluation of the clinical and videotape data. Each participant was examined for Essential Tremor by using the Washington-Heights-Inwood Genetic Study of ET criteria (WHIGET, 1995–2000) (14, 15). Each participant was rated for rest and postural tremors and was asked to perform a series of tasks (pouring water, drinking water from a cup, using a spoon, finger-to-nose movements, writing, and drawing spirals) to elicit action tremor. All tasks were performed with the dominant and non-dominant hand, except writing, and the severity of tremor was rated based on these tasks. Additional assessments included chin and head tremor, voice tremor, tandem gait, and presence of dystonia, with the involved body region specified when present. Limb bradykinesia was rated, and the examiner noted whether micrographia, hypomimia, or parkinsonian gait were present. Each participant was classified as having definite, probable or possible ET, and it was recorded whether they had a diagnosis of PD, dystonia or possible dystonia, myoclonus, or psychogenic tremor, or were considered normal. Participants were evaluated for features of PD using the diagnostic criteria of the UK Parkinson Society Brain Bank (16, 17). Diagnosis of PD required presence of bradykinesia plus at least one of muscular rigidity, resting tremor or postural instability (16).

### Whole genome sequencing (WGS) and genetic analyses

#### Whole genome sequencing

Genomic DNA was isolated from peripheral blood cells using standard methods. Libraries were prepared using the TruSeq DNA PCR-free kit (Illumina San Diego CA, United States). Paired end sequencing (2x150bp) was performed at >30x coverage per sample. Resulting libraries were sequenced on Illumina HiSeq TENx (Illumina San Diego, CA). Whole genome sequencing (WGS) was performed by the NY Genome Center. QC of data was performed and a mean target coverage of >30X reads was obtained for the sequence data and >75% of bases sequenced had a quality score of >Q30. BAM format files contain all passed filter reads and quality scores. Bioinformatic analysis of SNVs and indels was performed and included the following: (1) Alignment of raw reads to the human genome assembly GRCh37 (hg19) from the genome reference consortium using BWA-mem, (2) removal of duplicate reads using Picard MarkDuplicates, (3) local indel realignment and base quality score recalibration using the Genome Analysis Toolkit (GATK), (4) Variant calling using the GATK Haplotype caller, and (5) Joint genotyping using the tool GATK GenotypeGVCFs. The recalibrated variant calls in VCF format were annotated using snpEFF, allele frequencies from the 1,000 genomes project, NHLBI GO Exome Sequencing Project (ESP), Exome Aggregation Consortium (ExAC), dbSNP 142 rsIds, conservation scores from PhyloP, GERP, PhastCons, damaging effect predictions from Polyphen 2, SIFT, clinically relevant information from OMIM, ClinVar, regulatory potential scores from Regulome, gene ontology, pathway annotations from UniProt and ConsensusPathDB.

#### Autozygosity mapping

Shared regions of homozygosity (ROHs) > 5 Mb (runs of variants with >75% of variants homozygous that are >5 Mb) which are suggestive of parental relatedness and consanguinity were identified in two affected sibs (16–055 and 16–059) using the clinical reporter platform in opal (Fabric Genomics). ROHs were used to guide candidate gene identification.

#### SNV/indel analyses

To identify rare genic SNV/indels segregating with ET a family-based analysis was performed. SNV/indel filtration was performed using clinical reporter in opal (Fabric genomics) with the following conditions: inheritance-recessive or dominant; quality-coverage >10, quality>30 and genotype quality >20; allele frequency-1KG < 1%, EVS < 1%, ExAC <1%; Omicia Score-0.6-1.0. Fabric Genomics VAAST and Phevor algorithms were also used to evaluate variants. The VAAST algorithm ranks variants and their associated genes by their likelihood to cause disease (18). VAAST evaluates predicted impact on function, allele frequency as well as evolutionary conservation. The Phenotype Driven Variant ontological Reranking tool (Phevor) (19) was used to rerank prioritized genes using the following Human Phenotype Ontology (HPO) seed terms: HP:0030188, HP:003186, HP:0030185, HP:0002378, HP:0002346, HP:0002345, HP:0002322, HP:0002174, HP:0002080, HP:0001337, HP:0002451, HP:0002268, HP0001332, HP:0001304, HP:0007325, HP:0002548, HP:0001300.

#### Filtering using gene panels for ET, PD and other neurogenetic disorders

Gene panels were constructed to allow filtering based on known disease genes using the clinical reporter in Opal (Fabric Genomics). Gene panels (Supplementary Data) consisted of the following gene sets: (1) Known ET genes, (2) Known PD genes, (3) Known dystonia genes, (4) Known neurodegenerative disease genes, (5) Mouse tremor phenotype genes (the Jackson Laboratory), (6) Known Hereditary Spastic Paraplegia (HSP) genes, (7) Known Spinocerebellar Ataxia (SCA) genes, (8) K + channel genes, and (9) Ca2 + channel genes.

#### Prioritization of candidate genes

##### Databases

To prioritize candidate genes, we examined the function of genes with rare variants predicted to be deleterious and damaging. We assessed the documented neurodevelopmental, neurobehavioral or neurodegenerative phenotype in humans or in animal disease gene models (mouse, *Caenorhabditis elegans*, Drosophila, Zebrafish). For human annotation, we used published literature in Pubmed together with Genecards, the database for annotation, visualization and integrated discovery (DAVID) v6.7, the human phenotype ontology database, Phenotips, OMIM, HGMD, Orphanet and DECIPHER. For animal models we used published literature, Genecards, mammalian phenotype ontology, the Jackson laboratory, flybase, wormatlas, and the zebrafish model organism database.

##### Co-segregation of variants with ET within the family

Variants identified from the analysis that were annotated (genic coding and intronic splice variants) and/or predicted by *in silico* prediction tools to be deleterious or damaging (coding variants) were assessed for co-segregation with ET in the family. The criteria that we used to define co-segregation is as follows: (1) the annotated variant was present in all affected ET individuals and (2) absent from unaffected individuals within a family. Sanger sequencing was used to validate and confirm variants within a family and to genotype family members with available DNA that did not have WGS data.

### Functional studies

#### DNA constructs

Human cDNAs for the *PRPF40B* gene were obtained commercially (e.g., GE Dharmacon, Lafayette, Co). For expression and detection, a HA-epitope tag was introduced at the mature N-terminus after the signal sequence and cDNAs were inserted into the expression vector pcDNA3.1. The candidate variant was introduced using the Quick-change Lightning site directed mutagenesis kit (Agilent). To verify the correct introduction of the variant/mutation all DNA constructs were confirmed by Sanger sequencing.

#### Cell culture and transfections

Cells were transfected with Lipofectamine LTX with plus reagent (Thermofisher Scientific). Cells were analyzed 48–72 h after transfection.

#### Immunocytochemistry

For immunocytochemistry, cells were fixed with 4% paraformaldehyde in 100 mM sodium phosphate buffer, pH 7.2 containing 4% sucrose. Cells were permeabilized with 0.1% Triton and nonspecific binding sites were blocked with 10% goat serum. Primary antibodies to the candidate gene of interest, anti-HA-tag, and to other cellular structures or proteins as needed were used followed by secondary antibodies. Immunocytochemistry with an anti-*PRPF40B* antibody (AB122491; Abcam) Colocalization with an endosomal marker, anti-EEA1 antibody (AB70521; Abcam) was performed. Images were acquired using a scanning confocal microscope. Confocal images were assembled into figures using Adobe photoshop (Adobe Systems).

## Results

### Clinical features

**Figure 1:**
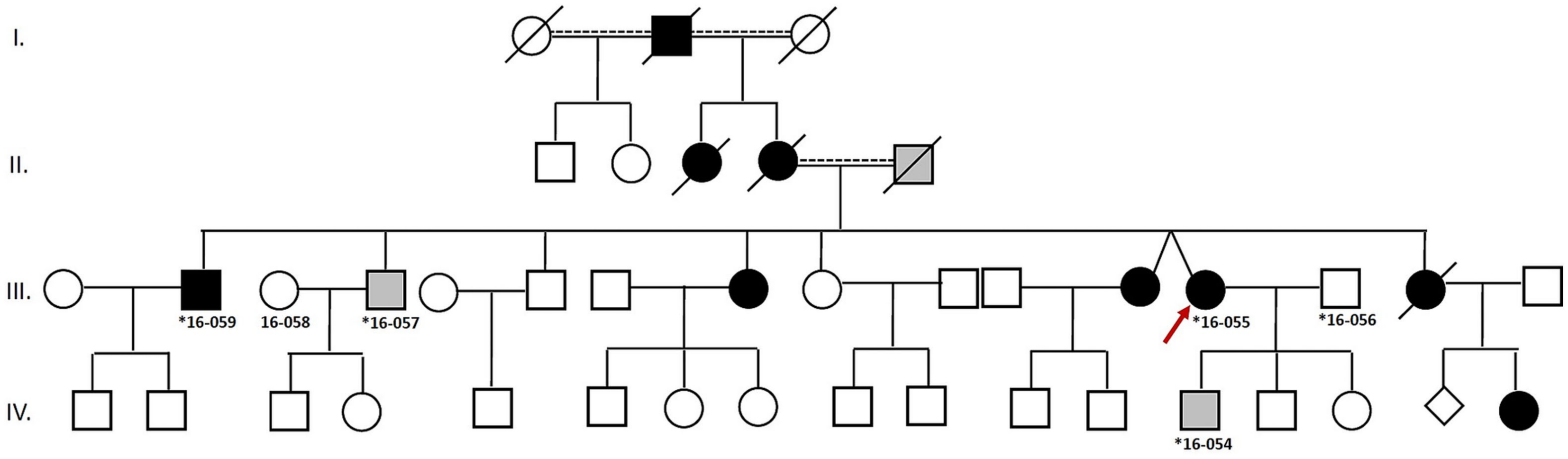
Pedigree of ET family TR-ETM-54. A four-generation pedigree of a Turkish family with ET. The proband is indicated by an arrow. Square symbols represent males, circles represent females, and diamond symbols represent individuals whose gender is unknown. Affected individuals are indicated by filled symbols and unaffected individuals by clear symbols. Affected individuals with black-filled symbols have a clinical diagnosis of severe or moderate ET. Affected individuals with grey-filled symbols have a clinical diagnosis of mild ET. Double horizontal relationship lines represent marriages with consanguinity/endogamy. Sequenced individuals are indicated by an asterisk.

The TR-ETM-54 family is a four-generation family from central Anatolia, with multiple family members affected with ET. Consanguineous marriages are common practice in the area. ET is known to have segregated in the family for generations. A pedigree of the family is shown in Figure 1. A total of 6 individuals from the family were clinically assessed (Table 1). A total of 4 family members were diagnosed with ET; two individuals also had a diagnosis of Parkinson’s Disease (PD) (16–054 and 16–055). The disease course was slowly progressive. The age of onset of affected family members ranged from 7 to 51 years. Two family members that were clinically assessed were unaffected.

**Table 1:** Clinical diagnosis and *PRPF40B* genotype of individuals from family TR-ETM-54.

Individual	Age at onset of tremor	Age at examination	Essential tremor	Parkinson’s disease	*PRPF40B* genotype
16–054	7	43	Mild	Present	Heterozygous
16–055	51	61	Severe	Present	Homozygous
16–056	-	63	Unaffected control	Unaffected control	Wildtype
16–057	Unknown	67	Mild	No	Heterozygous
16–058	-	60	Unaffected control	Unaffected control	N/A*
16–059	20	58	Moderate	No	Homozygous

*Not sequenced.

### Genetic analyses

WGS was performed using DNA from four affected individuals including a proband (16–055) and two siblings (16–059 and 16–057), and the son of the proband (16–054). An unaffected individual (16–056; a spouse, married-in and non-consanguineous) was also sequenced.

#### Autozygosity mapping

Autozygosity mapping was initially performed in the proband and an affected sibling (16–055 and 16–059) with severe/moderate ET. We identified seven chromosomal regions in the proband with ROH > 5 Mb (Table 2). Two of the ROH regions identified in the proband overlapped with ROHs in the affected sibling (16–059) on Chr1: 110246364–116,226,005 and Chr12: 46595701–54,330,441.

**Table 2:** Regions of homozygosity identified in the proband in the ET family TR-ETM-54.

Chromosome	Start	End	Length (Mb)	% homozygous
1	110,246,364	116,226,005	6	97.9
3	23,210,475	29,891,801	6.7	98.1
5	5,791,460	17,520,759	11.7	98.4
5	21,573,131	29,424,800	7.9	98.9
11	74,877,864	86,739,604	11.9	98.5
12	21,555,582	27,683,791	6.1	97.8
12	46,595,701	54,330,441	7.7	96.6

After filtering variants in the shared ROHs for quality, zygosity, population frequency, and predicted pathogenicity based on annotation, we prioritized a variant in *PRPF40B*. The genes and variants that were excluded from the analysis are summarized in the Supplementary Data.

#### Co-segregation analysis

*PRPF40B* is located in the 7.7 Mb ROH (Chr12: 46595701–54330441) identified in the proband and affected sibling in the family (Table 2). The *PRPF40B* genotype of sequenced individuals is summarized in Table 1. The *PRPF40B* variant, c.497C > T (p.Ser166Leu) was identified in the homozygous state in the affected proband (16–055) and sibling (16–059) (brother). A second sibling (16–057) (brother) and an affected child of the proband (16–054), both with mild tremor are heterozygous for the *PRPF40B* variant. An unaffected individual (16–056), the spouse of the affected proband (16–055), was wildtype for the *PRPF40B* variant.

#### *In silico* predictions

**Figure 2:**
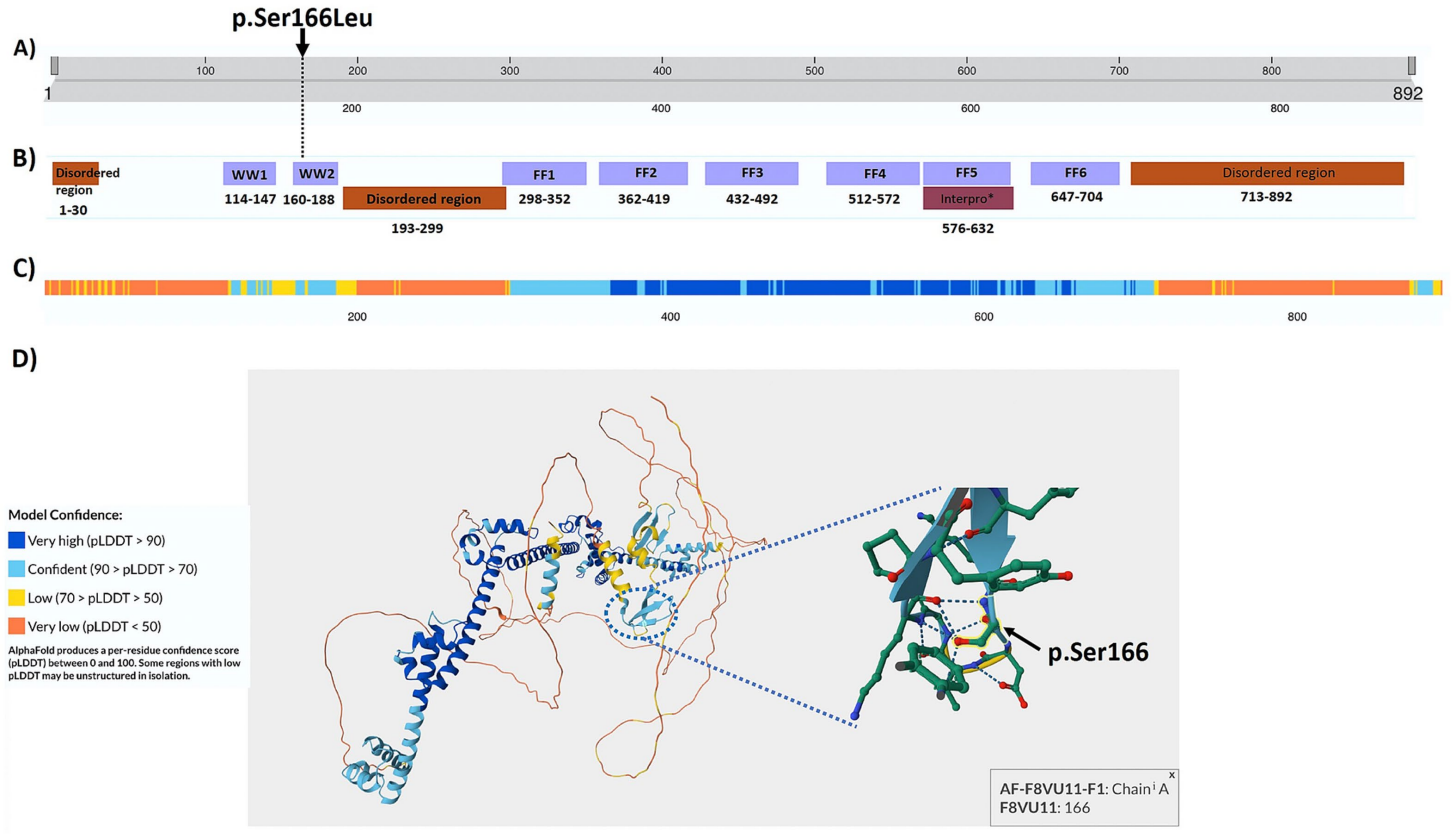
Protein structure and 3D model of PRPF40B. The protein structure, protein domains, and 3D ribbon protein model for PRPF40B, based on data from SWISS-MODEL for F8VU11 (PRPF40B_Human) and UniProt. An arrow indicates the position of the PRPF40B variant, p.Ser166Leu, identified in the ET family. **(A)** The amino acid positions within the PRPF40B protein **(B)** Known protein domains within PRPF40B. WW domain and FF protein domains are indicated. The PRPF40B variant, p.Ser166Leu, is located in the WW2 domain **(C)** PRPF40B model confidence scores **(D)** A 3D ribbon protein model for PRPF40B.

The *PRPF40B* variant (Chr12:50027247 C > T; c.497C > T, p.Ser166Leu) identified in this family has an allele frequency of 0.0004455 (126/282844 alleles; 0 homozygotes) in the genome aggregation database (gnomAD), is absent in the homozygous state in gnomAD and was absent from a Turkish population Genome Database (0/8,050 alleles). This variant has been reported as a secondary finding, a somatic variant with a variant allele frequency of 40%, in one individual with pure erythroid leukemia (20) (Genomenon Mastermind search engine; 30 million abstracts and 6 million genomic full text articles searched). The variant results in an amino acid substitution of a conserved amino acid (GERP: 4.5399) Serine to Leucine at amino acid 166. Multiple *in silico* variant prediction algorithms predict this variant to be deleterious and damaging to the structure and function of the protein: DANN (score: 0.9987), MutationTaster (Disease causing), Mutation assessor (Medium; score 3.12), FATHMM (Damaging; score −1.7), FATHMM-MKL (Damaging; score 0.9303), FATHMM-XF (Damaging; score 0.8859), LRT (Deleterious; score 0.00002999), DEOGEN2 (Damaging; score 0.7878, 0.7681), SIFT (Damaging; score 0.032, 0.013, 0.014), SIFT4G (Damaging; score 0.047, 0.048, 0.045), PROVEAN (Damaging; score −4.98, −4.5), PrimateAI (Damaging; score 0.8073), MetaSVM (Damaging; score 0.4141) and MetaLR (Damaging; score 0.6993). The *PRPF40B* gene has a very low rate of Loss of Function (LOF) (Z score = 5.895) variation, consistent with autosomal recessive disease and suggesting that this gene is highly constrained and intolerant to LOF variants. The variant (c.497C > T, p.Ser166Leu) is predicted to affect the main biologically relevant transcript NM_001031698.3. *PRPF40B* is ubiquitously expressed (data source GTEx analysis release V10 dbGaP Accession phs000424.v10.p2.) and in the CNS, highest levels of expression are observed in the cerebellum and in non-CNS tissues in the thyroid and testis. The *PRPF40B* p.Ser166Leu variant is located within a conserved region of the WW protein domain (Figure 2).

#### Classification of the variant according to ACMG/AMP criteria

Classification of the variant according to the standards and guidelines of the American College of Medical Genetics (ACMG) and the Association for Molecular Pathology (AMP) criteria suggests the *PRPF40B* variant (c.497C > T, p.Ser166Leu) is a variant of uncertain significance (VUS). The following ACMG/AMP criteria were used to classify the variant as a VUS: (1) Functional data: PM1 pathogenic moderate. The *PRPF40B* variant, p.Ser166Leu, is located in a well-established functional domain, the WW protein domain that mediates protein–protein interactions. (2) Population data. PM2 pathogenic moderate. The variant is at an extremely low allele frequency for a recessive disease and is absent in the homozygous state in in the GnomAD database and a Turkish population database. (3) Rate of benign missense variants. PP2 supporting evidence. The *PRPF40B* variant is a missense variant in a gene that has a low rate of benign missense variants (1 benign/156 variants). (4) *In Silico* Predictions. PP3 supporting evidence. Multiple lines of computational evidence support a deleterious and damaging effect on the gene product.

#### Analysis of additional ET cohorts

To identify additional ET families with *PRPF40B* variants, we analyzed 166 Turkish ET cases and 104 multi-generational ET families with European ancestry from the Family Study of Essential Tremor (FASET) (dbGaP study accession: phs000966.v3.p2). Additional ET cases or families with *PRPF40B* variants were not identified.

### Functional studies of *PRPF40B*

**Figure 3:**
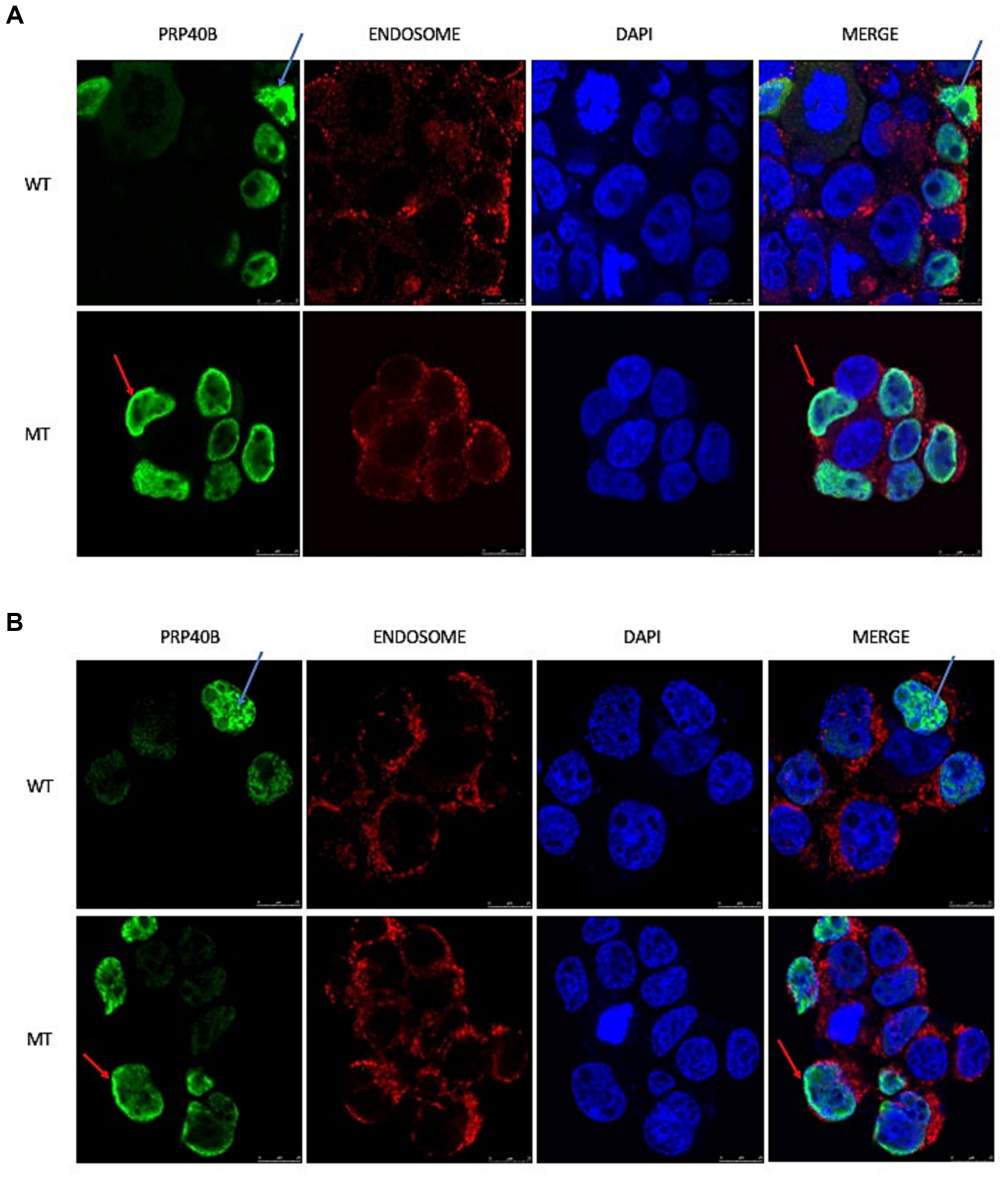
Expression of mutant PRPF40B in HEK293T cells. To determine whether the variant identified in PRPF40B effects the expression, localization, and function of the PRPF40B protein, PRPF40B was expressed in HEK293T cells and analyzed using immunocytochemistry with an anti-PRPF40B antibody (AB122491; Abcam) and confocal microscopy. Colocalization with an endosomal marker, anti-EEA1 antibody (AB70521; Abcam) was also performed. Wildtype (WT) PRPF40B localizes to splicing factor-rich nuclear speckles in the nuclear compartment (**A,B**; blue arrow). For mutant (MT) PRPF40B (c.497C > T, p.Ser166Leu), we observed enrichment and localization to the nuclear membrane (**A,B;** red arrow). Representative images are shown in **A,B**. Replicates for each experiment was performed. Quantification was not performed.

To determine whether the variant identified in *PRPF40B* effects the expression, localization and function of the *PRPF40B* protein, *PRPF40B* was expressed in HEK293T cells and analyzed using immunocytochemistry with an anti-*PRPF40B* antibody (AB122491) and confocal microscopy. Wildtype *PRPF40B* localizes to splicing factor-rich nuclear speckles in the nuclear compartment (Figure 3; blue arrow). For mutant *PRPF40B* (c.497C > T, p.Ser166Leu), we observed enrichment and localization to the nuclear membrane (Figure 3; red arrow). Representative images are shown in Figure 3.

## Discussion

Clinical evaluation and whole genome sequencing of affected and unaffected family members of family TR-ETM-54 identified *PRPF40B* as a candidate gene for ET. Both homozygosity and heterozygosity for the *PRPF40B* p.Ser166Leu allele were observed in affected family members. The variant was classified as a VUS according to ACMG/AMP criteria. Further functional studies will be needed to better understand its causal relevance and pathogenicity.

The proband and a sibling homozygous for the variant expressed a more severe phenotype, and the proband had previously received a clinical diagnosis of Parkinson’s disease (PD) in addition to ET during the initial evaluation in Turkey. The son of the proband was also diagnosed with PD (and ET). However, drug-induced Parkinsonism cannot be ruled out in this individual, as he had been taking anti-psychotic medication (800 mg quetiapine, 15 mg haloperidol, 20 mg aripiprazole, and 5 mg trifluoperazine) since age 22. Additional clinical studies including DaTscan imaging will be needed to confirm the diagnosis of PD. The combination of ET and Parkinsonian features in these individuals could represent coincidental idiopathic PD, which is relatively common, or may be better conceptualized within the ET-plus framework, a recognized phenotype in which additional signs (including mild Parkinsonism) may emerge over time.

*PRPF40B* is the mammalian homolog of the U1 snRNP-associated yeast splicing factor Prp40. The first stable complex formed during the assembly of spliceosomes onto the pre-mRNA substrate in mammals includes the U1 snRNP, which recognizes the 5′ splice site and the splicing factors SF1 and U2AF, which bind to the branch point sequence, polypyrimidine tract, and 3′ splice site. Collectively termed the spliceosome, pre-mRNA splicing is a multicomponent splicing machinery in the nucleus. *PRPF40B* is highly enriched in speckles, colocalizes with splicing factors and binds SF1 and U2AF in the cell nucleus (21). The importance of proper nuclear speckle function is highlighted by the large number of genetic disorders caused by mutations in genes encoding nuclear speckle proteins (22). In the Online Inheritance in Man (OMIM) database, a total of 291 out of 380 genes, whose encoded proteins localize within nuclear speckles, based on The Human Protein Atlas database, were found to be associated with a human disease phenotype. The most common clinical features and associated HPO terms indicate an enrichment of neurological symptoms, indicating the importance of nuclear speckles during brain development (22).

Clinical and post mortem studies have implicated the cerebellum in the pathogenesis of ET. Alternative splicing in the brain generates a diverse transcript repertoire, and brain regions such as the cerebellum may be more susceptible to the effects of abnormal nuclear speckle proteins and mis-splicing events. Interestingly, *PRPF40B* has been implicated in the pathogenesis of other neurological disorders with tremor, including Huntington’s disease (23, 24) and Rett syndrome (25). Moreover, *PRPF40B* is a known WW binding domain partner of HTT protein (24) in Huntington’s disease and MECP2 protein (25) in Rett syndrome. Further functional studies are needed to validate the connection between *PRPF40B*, HTT, MeCP2, and other WW domain proteins (e.g., HYPA and HYPB), as well as splicing in nuclear speckles in the cerebellum and relevance to the pathogenesis of ET.

To our knowledge, *PRPF40B* represents the first essential tremor candidate gene whose product is a nuclear speckle–localized splicing factor; previously described ET genes and loci have more commonly involved other pathways, such as mitochondrial proteases, RNA-binding proteins, and genes involved in neuronal connectivity and cerebellar signaling (26, 27).

In summary, we have identified a nuclear speckle protein, *PRPF40B*, as a candidate gene in a Turkish family with tremor. This consanguineous family illustrates that leveraging population endogamy and autozygosity mapping, together with video-based clinical assessments, can be a productive strategy for identifying rare candidate genes for ET. We acknowledge that a limitation of the study is that only 6 family members were clinically assessed. Further studies are needed to clinically assess additional family members and identify additional families with *PRPF40B* mutations. Given the importance of nuclear speckle function for neuronal development, these proteins may represent therapeutic targets during brain development for neurological disorders. More broadly, our findings raise the possibility that perturbation of nuclear speckle–associated splicing factors contributes to ET susceptibility and related phenotypes, and that this pathway may ultimately help to define new mechanistic targets for therapeutic development in neurological disease.

## Data Availability

Genotype and WGS data is publicly available and was submitted to NCBI dbGaP with study accession number phs001507.v2.p2 Columbia-Yale-Bilkent Study: Genetic Study of Essential Tremor. Genotype, Phenotype, and WGS data for the Family (TR-ETM-54) included in this publication were not submitted to NCBI dbGaP (study accession number phs001507.v2.p2), due to legal, ethical and privacy restrictions of sharing the data from this family. Requests to access the dataset for this family should be directed to Tayfun Ozcelik (email: tozcelik@bilkent.edu.tr).
