# Supplementary material for: A homozygous missense variant, p.Ser166Leu in the PRPF40B gene, in a 7.7 Mb region of homozygosity in a consanguineous Turkish family with essential tremor
Source: Front Neurol. 2026 May 26;17:1796664. doi: 10.3389/fneur.2026.1796664 (PMC13247433; doi:10.3389/fneur.2026.1796664)
Supplement: Supplementary file 1 [file Data_Sheet_1.PDF]

Exclusion of variants in ROHs

Autozygosity mapping was initially performed in the two affected siblings (16-055 and 16-059) with severe/moderate ET. As described in Table 2, we identified 7 chromosomal regions in the proband with ROH > 5 Mb. Two of the ROH regions identified in the proband overlapped with ROHs in the sibling (16-059): Chr1: 110246364-116226005 and Chr12: 46595701-54330441. SNV/indel filtration with the following conditions was performed in each ROH to identify candidate genes/variants:

1. Allele frequency: 1KG<1%, EVS<1%, ExAC<1%.
2. The variant is homozygous in the affected proband16-055 and affected sibling 16-059
3. The variant passed quality, GQ and coverage filters
4. The variant is in a coding region/gene

After identifying the shared ROHs in 16-055 and 16-059 and filtering variants, 3 genes met the above criteria. The genes and variants are summarized in the Table below and reasons for exclusion/inclusion described here:

1. A missense variant in *CSF1* (rs113687620; p.Met215Thr) was excluded because *in silico* predictions predict that this variant is a neutral tolerated variant and is not predicted to be damaging or deleterious to the structure and function of the *CSF1* protein.

2. The indel variant in *DENND2C* (rs562839824) is located in a homopolymer stretch. Insertions and deletions on homopolymers are a significant source of erroneous variant calls (sequencing artefact); coupled with the high allele frequency in GnomAD we excluded the variant.

3. After excluding the variants in *CSF1* and *DENND2C*, the only remaining candidate was *PRPF40B*, and therefore we prioritized the variant in *PRPF40B*. The missense variant in *PRPF40B* (rs146358365; p. Ser166Leu) is rare with an allele frequency of 0.0004455 (126/282844 alleles; 0 homozygotes) and is absent in the homozygous state in the genome aggregation database (gnomAD. Multiple *in silico* variant prediction algorithms predict this variant to be deleterious and damaging to the structure and function of the protein. In the CNS PRPF40B shows highest levels of expression in the cerebellum. Further details of the analysis of the variant and gene that we performed is described in the manuscript in the methods and results.

Gene Name	Position dbSNP	Nt. Change	Effect	Proband zygosity (16-055)	Sibling 1 zygosity (16-59)	Quality, GQ, Coverage	Varsome	*In silico* Varsome predictions	GnomAD
CSF1	chr1:110465887 , rs113687620	T → C, c.644T>C, p. Met215Thr	missense	Homozygous	Homozygous	1941, 93, 31:0:31	https://varsome.com/variant/ hg19/1-110465887-T-C	Mutation taster=polymorph, SIFT score 1-tolerated, provean score -0.3, 0.02, -0.49 -neutral, DANN score 0.2099, Gerp:4.33	Pass for exomes and genomes, Total allele frequency=0.00009871
DENND2C	chr1:115197384 , rs562839824	T → TA, c.-573-3dupT	Insertion in homopolymer region	Homozygous	Homozygous	2726,64, 24:1:23	https://varsome.com/variant/ hg19/1-115197384-T-TA	NA	Pass for exomes and genomes, Total allele frequency=0.06777
PRPF40B	chr12:50027247 ,rs146358365	C → T, c.497C>T ,p.Ser166Leu	Missense	Homozygous	Homozygous	2669,99,36:0:36	https://varsome.com/variant/ hg19/12-50027247-C-T	Mutation taster=disease causing, SIFT score 0.013-damaging, provean score -4.98 , -4.5, -damaging DANN score 0.9987, Gerp:4.5399	Pass for exomes and genomes, Total allele frequency=0.0004305

Exclusion of Variants in known ET, PD and Related Disease Genes

Gene panels were constructed to allow filtering based on known disease genes. The genes that were evaluated in these panels are provided as supplementary data. SNV/Indel filtration was then performed with conditions as described in the paper. The following variants were excluded:

A heterozygous variant located in intron 7 of *EIF4G1*, c.559-6C>A, which is not located in a canonical splice site, and is therefore not predicted to effect splicing was identified in the proband and other affected family members.

Two heterozygous variants were identified in *VPS13A* in all affected family members:

1) One of the variants, VPS*13A* (NM_033305.3); c.5594C>T (p. Thr1865Ile) is rare in the population databases with an allele frequency of f = 0.000478 in gnomAD genomes. *In Silico* predictions were benign supporting or uncertain and the variant is not predicted to impact the structure/function of the protein. https://varsome.com/variant/hg19/rs143449578?annotation-mode=germline
2) The second variant in *VPS13A* (NM_033305.3): c.4927G>A, p.(Asp1643Asn) is also predicted to be benign (BP4 benign strong) based on in silico predictions and the allele frequency in gnomAD (f = 0.00442).

No other candidate variants were identified in known ET, PD, or related disease genes.
